# Role of chitinase-3-like protein 1 in liver diseases: A comprehensive review

**DOI:** 10.1016/j.gendis.2025.101653

**Published:** 2025-04-24

**Authors:** Chao Tian, Shizhou Deng, Ming Yang, Baochen Bai, Lai Wei

**Affiliations:** a Hepatopancreatobiliary Center, Beijing Tsinghua Changgung Hospital, School of Clinical Medicine, Tsinghua Medicine of Tsinghua University, Beijing 102218, China; b Ministry of Education Key Laboratory of Digital Intelligence Hepatology, Tsinghua University, Beijing 100084, China; c Research and Development Department, Guangdong Longsee Biomedical Corporation, Guangzhou, Guangdong 510700, China; d Department of Cardiology, Peking University People's Hospital, Beijing 100044, China

**Keywords:** Chitinase-3-like protein 1, Hepatic fibrosis, Hepatocellular carcinoma, Liver disease biomarker, Non-alcoholic steatohepatitis, Signaling pathways

## Abstract

Chitinase-3-like protein 1 (CHI3L1) is part of the glycoside hydrolase family 18. Despite lacking enzymatic activity, its unique structure allows it to bind to ligands, altering its steric configuration to mediate cell proliferation, inflammation, fibrosis, and carcinogenesis. In liver disease, CHI3L1 serves as a common diagnostic biomarker for hepatitis-related fibrosis. Additionally, CHI3L1 can predict the risk of non-alcoholic steatohepatitis, the progression of hepatic fibrosis, and the prognosis of alcoholic liver disease and hepatocellular carcinoma. It also aids in diagnosing and staging non-alcoholic fatty liver disease-related and alcoholic liver disease-related fibrosis, and in monitoring hepatitis-related fibrosis treatment. Furthermore, CHI3L1 is secreted by various cells, including hepatocytes, hepatic stellate cells, macrophages, and mesenchymal stem cells, to regulate hepatic injury, fibrosis, steatosis, and hepatocellular carcinoma through different signaling pathways. This review highlights CHI3L1's dual roles as both a biomarker and regulator in various liver diseases, aiming to broaden researchers' understanding of its potential applications.

## Introduction

Chitinase 3-like protein 1 (CHI3L1 or YKL-40), a chitinase-like protein (CLP), is part of the glycoside hydrolase family 18. It is encoded by genes located on chromosome 1 in humans and mice. Interestingly, other homologous proteins encoded by the same domain include breast regression protein 39 (BRP-39), YKL-40, and human cartilage glycoprotein 39 (HcGP-39, GP39). The term used for this protein in studies depends on its cellular or species source.^1^ To avoid confusion in expression, we will uniformly use “CHI3L1” throughout the following review.

CHI3L1 can be secreted by various cells, including chondrocytes, synoviocytes, macrophages, neutrophils, fibroblasts, endothelial cells, epithelial cells, and tumor cells.^1^, ^2^, ^3^ Notably, CHI3L1 is enriched in the liver compared with other tissues.^4^ This makes CHI3L1 an ideal biomarker for liver diseases. Additionally, CHI3L1 can be regulated by various cytokines^5^^,^^6^ and noncoding RNAs^7^, ^8^, ^9^ to influence pathophysiological processes such as cell proliferation,^10^ immune response,^11^ inflammation,^12^ extracellular matrix production,^13^ and carcinogenesis,^14^^,^^15^ impacting the development of both cancerous and non-cancerous diseases.^16^

In detail, CHI3L1 has been shown to play a crucial role in modulating immune responses and inflammation. It can contribute to macrophage differentiation and dendritic cell accumulation.^17^ Furthermore, CHI3L1 is involved in tissue remodeling through its interaction with the extracellular matrix, which is essential for processes such as wound healing and tumorigenesis.^18^ Moreover, CHI3L1 has been linked to cell survival and apoptosis regulation, particularly under stress conditions such as hypoxia and oxidative stress, which are commonly observed in ischemia-reperfusion conditions^19^ and tumor microenvironments.^20^ These diverse functions underscore the multifunctional nature of CHI3L1 in both normal physiology and disease pathogenesis. CHI3L1 has been proven to be involved in asthma,^21^ lung fibrosis,^22^ atherosclerosis,^23^ Alzheimer's disease,^24^ and various cancers such as colon cancer,^25^ glioblastoma,^26^ prostate cancer,^27^ and cervical cancer.^28^

In this review, we comprehensively summarize the application of CHI3L1 and CHI3L1-based models as biomarkers for various liver diseases. Additionally, we explore the CHI3L1-mediated pathways in liver disease pathology, aiming to enhance researchers' understanding of CHI3L1 as biomarkers and therapeutic targets.

### CHI3L1 as a biomarker for different liver diseases

The clinical value of CHI3L1 has been preliminarily confirmed through systems biology.^4^^,^^29^^,^^30^ It has been tested as a common serum marker for various etiologies of hepatic fibrosis.^31^, ^32^, ^33^, ^34^ Currently, CHI3L1 is recommended for diagnosing hepatic fibrosis or cirrhosis in Chinese clinical guidelines.^35^^,^^36^ Additionally, it is being explored for predicting, diagnosing, and monitoring other liver diseases.

### CHI3L1 as a biomarker for hepatitis related fibrosis

Chronic hepatitis B (CHB) and chronic hepatitis C (CHC) are common chronic liver diseases, particularly in low- and middle-income countries.^37^ It is estimated that about 295.9 million people are infected with hepatitis B virus (HBV) and 57.8 million are infected with hepatitis C virus (HCV) in the world.^38^ Without screening and control, both CHB and CHC can progress to hepatic fibrosis, cirrhosis, and even hepatocellular carcinoma (HCC).^39^^,^^40^ Guidelines on HBV therapy suggest initiating antiviral treatment for CHB with significant fibrosis (≥F2) to prevent disease progression.^41^ Therefore, early diagnosis of CHB- and CHC-related fibrosis, detection of fibrosis progression, and monitoring the effectiveness of antiviral treatment are crucial.

Liver biopsy is the gold standard for diagnosing liver fibrosis, but it is invasive, prone to sampling errors, and not suitable for frequent use due to low patient compliance.^42^ Transient elastography is a non-invasive tool to identify progressive liver fibrosis and early cirrhosis by measuring liver stiffness. It has been approved for diagnosing hepatic fibrosis in America, Europe, and some Asian countries.^43^^,^^44^ However, its results can be affected by liver inflammation, necrosis, cholestasis, and severe steatosis.^45^ Transient elastography may also be unavailable in resource-limited settings. Serum markers (*e.g.*, hyaluronic acid (HA), laminin, and matrix metalloproteinase (MMP)) and parameters (*e.g.*, fibrosis score 4 (FIB-4) and aspartate aminotransferase to platelet ratio index (APRI)) have been studied as potential biomarkers for CHB- and CHC-related fibrosis, but they lack uniform cutoff values, and their diagnostic accuracy is not always satisfactory.

CHI3L1 has emerged as a promising alternative biomarker for diagnosing, detecting the progression, and assessing the treatment response of liver fibrosis caused by hepatitis. In CHB-related fibrosis, serum CHI3L1 levels are elevated and positively associated with fibrosis stages.^46^^,^^47^ Jiang et al^48^ conducted a prospective study involving three groups of patients: CHB, CHB-related fibrosis, and CHB-related HCC. They found that serum CHI3L1 levels were closely related to the severity of CHB, with the highest level for HCC (245.9 ± 189.55 ng/mL) and the lowest for CHB (81.11 ± 86.17 ng/mL). The diagnostic efficacy for significant fibrosis (F2, F3) in CHB was 0.97, as indicated by the area under the receiver operating characteristic curve (AUROC), outperforming known fibrosis markers (FIB-4_AUROC_ = 0.729, APRI_AUROC_ = 0.688). Similarly, Li et al^49^ confirmed that CHI3L1 could diagnose HBV-induced liver fibrosis and differentiate mild (F0–F1) from significant/advanced fibrosis (F2–F4). Notably, CHI3L1 has a higher diagnostic efficacy for CHB with advanced fibrosis (≥F3), with greater accuracy compared with other fibrosis markers (CHI3L1_AUROC_ = 0.988 > HA_AUROC_ = 0.958 > type IV collagen _AUROC_ = 0.95 > type III procollagen _AUROC_ = 0.846 > laminin _AUROC_ = 0.658).^4^ However, a study on children with CHB indicated that CHI3L1 could not differentiate mild from advanced fibrosis.^50^

Researchers have explored combining different markers to establish a diagnostic model that overcomes the limitations of using a single marker for diagnosing CHB-related fibrosis. Diagnostic models based on CHI3L1 have been constructed. One model, comprising CHI3L1, platelet count, and alpha-fetal protein (AFP), effectively detects HBV-related significant liver fibrosis with an AUROC of 0.805 and 0.819 in training and validation groups, respectively.^51^ Similarly, the CHI3L1 model, comprising CHI3L1, aspartate aminotransferase (AST), platelet count, and HA, has an AUROC of 0.801 in the entire group (AUROC of 0.786 in the training group and 0.831 in the validation group) for detecting significant fibrosis with mild hepatic injury.^52^

For CHC-related fibrosis, CHI3L1 levels correlate with the severity of HCV-related fibrosis.^53^ CHI3L1 has been reported as a diagnostic marker for hepatic fibrosis induced by HCV infection in kidney transplant patients, but it is inferior to another non-invasive biomarker, HA.^54^ Additionally, CHI3L1 can accurately diagnose early stages of HCV-related liver fibrosis and distinguish between different stages of fibrosis.^55^^,^^56^ However, several studies indicate that routine fibrosis testing is superior to CHI3L1 for diagnosing or staging HCV-related fibrosis.^57^ Furthermore, diagnostic models using fibrosis markers other than CHI3L1 outperform CHI3L1 in diagnosing HCV-related fibrosis.^58^^,^^59^

Although CHI3L1 may not be superior for diagnosing HCV-related fibrosis compared with other markers, it can monitor the progression of HBV- and HCV-related fibrosis^60^, ^61^, ^62^ and evaluate the effectiveness of antiviral therapy.^63^, ^64^, ^65^ Notably, CHI3L1 can be secreted by HCV-induced steatotic hepatocytes, and the CHI3L1/AST ratio can diagnose grade II and III steatosis caused by HCV.^66^ Information on the cutoff values and diagnostic efficacy of CHI3L1 for CHB- and CHC-related fibrosis is presented in Table 1.

**Table 1:** CHI3L1 as the biomarker for hepatitis-related fibrosis.

Disease	Aim	Cutoff value	Efficacy	Comparison with other biomarkers	Ref.
CHB with advanced fibrosis	Diagnosis	Cutoff value = 78.48 ng/mL	sensitivity = 0.918; specificity = 0.917;	**CHB with advanced fibrosis:** CHI3L1 (AUROC = 0.988) > HA (AUROC = 0.958) > type IV collagen (AUROC = 0.95) > type III procollagen (AUROC = 0.846) > laminin (AUROC = 0.658)	4
CHB with significant fibrosis	Diagnosis	Cutoff value = 68.75 ng/mL;	sensitivity = 0.952; specificity = 0.890	**CHB with significant fibrosis (F2–F3 vs F0–F1):** CHI3L1(AUROC = 0.970)>(liver stiffness measurement (LSM)) (AUROC = 0.823) > FIB-4 (AUROC = 0.729) > APRI (AUROC = 0.688)	48
CHB with significant fibrosis	Diagnosis	Cutoff value = −1.90;	sensitivity = 0.732; specificity = 0.778	The CAP model (composed of CHI3L1, AFP and PLT) (AUROC = 0.804) > FIB-4 (AUROC = 0.701) > CHI3L1 (AUROC = 0.691) > APRI (AUROC = 0.639)	51
CHB with significant fibrosis	Diagnosis	Cutoff value = −0.56	Sensitivity: 0.717; Specificity: 0.729; PPV: 0.617; NPV: 0.809;	**Training group:** CHI3L1 model (composed of AST, PLT, HA and CHI3L1) (AUROC = 0.786) > Forns' index (AUROC = 0.753) > APRI (AUROC = 0.736) > FIB-4 (AURCO = 0.735) > Hui model (AUROC = 0.734)	52
CHC with significant fibrosis in kidney transplantation	Diagnosis	Cutoff value = 105 ng/mL;	NPV = 0.36;	**CHC with significant fibrosis:** HA (AUROC = 0.765) > CHI3L1 (AUROC = 0.615)	54
CHC with different stages of fibrosis	Diagnosis	NA		**CHC with significant fibrosis (≥F2):** CHI3L1 (AUROC = 0.802) > TE (AUROC = 0.798) > other serum biomarkers (HA, laminin, C-terminal procollagen I peptide, MMP-9, TIMP-1, TIMP-2 and MMP-9/TIMP-1 complex); **CHC with advanced fibrosis (≥F3):** TE (AUROC = 0.880) > CHI3L1 (AUROC = 0.798) > other serum biomarkers; **CHC with cirrhosis:** TE (AUROC = 1) > CHI3L1(AUROC = 0.998) > other serum biomarkers	55
CHB and CHC with significant fibrosis and cirrhosis	Diagnosis	**Significant fibrosis from moderate fibrosis (F2–F3 vs F0–F1):** Cutoff value = 90.00 ng/mL; **Cirrhosis from moderate fibrosis (F4 vs F0–F1):** Cutoff value = 112.00 ng/mL; **Cirrhosis from significant fibrosis (F4 vs F2–F3):** Cutoff value = 180.00 ng/mL;	Significant fibrosis from moderate fibrosis (F2–F3 vs F0–F1): sensitivity = 1.000; Specificity = 0.974; PPV = 0.909; NPV = 1.000; Cirrhosis from moderate fibrosis (F4 vs F0–F1): sensitivity = 0.981; specificity = 0.987; PPV = 0.981; NPV = 0.987; Cirrhosis from significant fibrosis (F4 vs F2–F3): sensitivity = 0.945; Specificity = 0.882; PPV = 0.963; NPV = 0.832;	**Significant fibrosis from moderate fibrosis (F2–F3 vs F0–F1):** CHI3L1 (AUROC = 0.98) > FIB-4 (AUROC = 0.65) > APRI (AUROC = 0.56) **Cirrhosis from moderate fibrosis (F4 vs F0–F1):**CHI3L1 (AUROC = 0.99) > FIB-4 (AUROC = 0.78) > APRI (AUROC = 0.76); **Cirrhosis from significant fibrosis (F4 vs F2–F3):** CHI3L1(AUROC = 0.95) > APRI (AUROC = 0.75) > FIB-4 (AUROC = 0.70)	56
ESRD-HCV infection related significant fibrosis	Diagnosis	Cutoff value = 9.60	sensitivity = 0.530; specificity = 0.910; PPV = 0.650; NPV = 0.860;	Regression model (AUC = 0.798) > APRI (AUROC = 0.787) > HA (AUROC = 0.650) > CHI3L1 (AUROC = 0.607)	58
CHB with significant fibrosis	Diagnosis and progression	Cutoff value = 76.00 ng/mL	specificity = 0.756; sensitivity = 0.591	**CHB with significant fibrosis:** CHI3L1(AUROC = 0.728) > FIB-4 (AUROC = 0.631) > APRI score (AUROC = 0.583)	61
CHC with moderate and advanced fibrosis	Diagnosis and treatment response	Cutoff value = 186.40 ng/mL	sensitivity = 0.800; specificity = 0.810; PPV = 0.800; NPV = 0.790;	**Fibrosis from mild stage of fibrosis (F2–F4 vs F0-1):** CHI3L1 (AUROC = 0.809) > HA (AUROC = 0.805) > PIIIP (AUROC = 0.747) > type IV collagen (AUROC = 0.742)	64

### CHI3L1 serves as a biomarker for NAFLD

Non-alcoholic fatty liver disease (NAFLD) is the most common chronic liver disease, affecting about 30.2 % of the global population.^67^ NAFLD encompasses non-alcoholic fatty liver (NAFL) and non-alcoholic steatohepatitis (NASH). If left untreated, NASH can lead to liver cirrhosis and even HCC.^68^ Experts have recently renamed NAFLD as metabolic dysfunction-associated liver disease to emphasize the importance of metabolic factors in this condition and to avoid excluding the possibility of alcohol consumption when making a diagnosis.^69^

A definitive diagnosis of NAFLD is established by liver biopsy, but this method has several limitations as mentioned above. Serum biomarkers have emerged as a promising alternative. Currently, numerous serum biomarkers are being studied for their potential in diagnosing NASH,^70^ with cytokeratin 18 (CK-18) being one of the most extensively studied. CK-18 fragments, released during caspase 3-mediated hepatocyte apoptosis, can be detected in serum via immunoassay. Since Feldstein et al^71^ first reported that serum CK-18 levels could predict NASH, a few studies with small population samples have confirmed these findings.^72^, ^73^, ^74^ However, CK-18 has limitations, including the lack of clinical trials with high levels of evidence, and its sensitivity and specificity are limited at the individual level. Furthermore, varying cutoff values proposed in different studies lead to significant heterogeneity, complicating the choice of threshold.

To overcome these limitations, researchers have combined CK-18 with other biological parameters, such as in the Nice model.^75^ Other predictive models combining clinical and laboratory parameters, like HAIR score,^76^ Palekar score,^77^ Gholam score,^78^ and NashTest score,^79^ have also been proposed. However, these models include relatively few participants and primarily focus on obese patients.^75^^,^^80^, ^81^, ^82^ Recently, epigenetic methods have suggested single-nucleotide polymorphisms of patatin-like domain 3,1-acylglycerol-3-phosphate O-acyltransferase (PNPLA3), NASH score,^83^ and non-coding RNAs like miR-122 and miR-192.^84^^,^^85^ However, no single serum marker or model can accurately distinguish NASH from NAFL with high sensitivity and specificity.

CHI3L1 can be used to predict, diagnose, and evaluate the severity and response to treatment of NAFLD or related fibrosis. Researchers have combined CHI3L1 with other parameters to enhance its predictive ability. One model, the NIS4® model, includes CHI3L1, miR-34a-5p, alpha-2-macroglobulin (A2M), and glycated hemoglobin (HbA1c). This model aims to predict NASH at risk and guide screening for populations susceptible to NASH. The AUROC of this model is 0.80, with a low cutoff value of 0.36 and a negative predictive value of 0.82, and a high cutoff value of 0.63 with a positive predictive value of 0.73.^86^ Harrison et al^87^ improved this model using parameters including miR-34a-5p, CHI3L1, sex, and sex∗miR-34a-5p to create the novel NIS2_+_™ model. This model is unaffected by age, sex, body mass index, and diabetes mellitus status and improves the performance of the NIS4® model. The AUROC of the modified model, NIS2_+_™, is higher than that of the NIS4® model (NIS2_+_™ _AUROC_ = 0.813 > NIS4® _AUROC_ = 0.792).

In the aspect of diagnosing NAFLD, CHI3L1 has been identified as a potential biomarker for NAFLD and diabetes related liver fibrosis. However, many studies found that CHI3L1 alone may not be as effective as other single biomarkers like CK-18 or sSiglec-7 in diagnosing NAFLD-related fibrosis. However, when CHI3L1 combined with other parameters such as waist-to-height ratio, HA, procollagen–III–peptide, CK-18 neoepitope M65, and type IV collagen 7s, to established different diagnostic models, they are quite effective for NAFLD with significant fibrosis (≥F2) and advanced fibrosis (≥F3), the AUROC of each model is more than 0.80 with different cutoff values owing to the different formulas.

**Figure 1:**
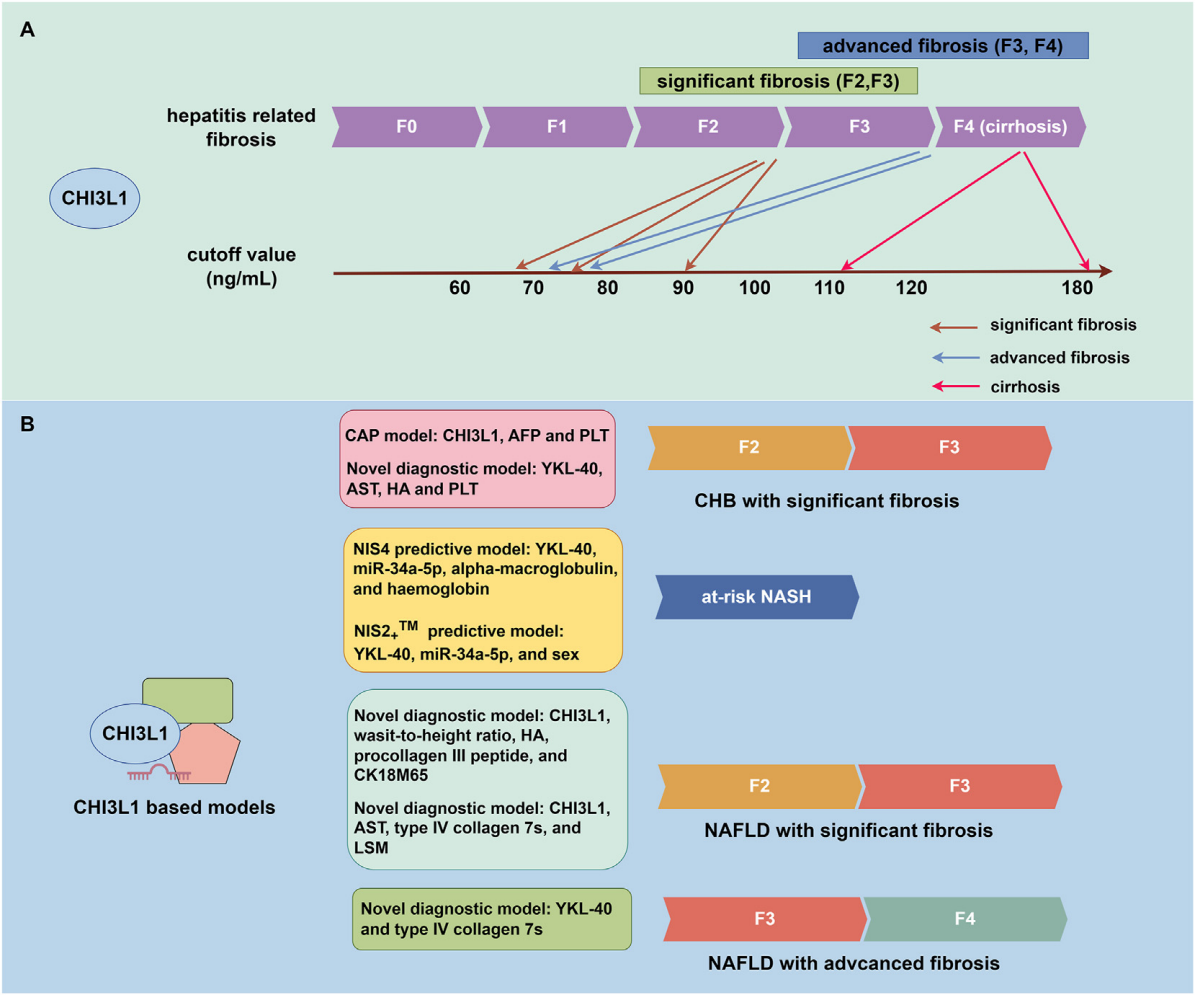
The diagnostic and predictive roles of CHI3L1 and CHI3L1-based models. **(A)** CHI3L1 can diagnose and stage various degrees of hepatitis-related fibrosis, including significant fibrosis (F2, F3), advanced fibrosis (F3, F4), and cirrhosis (F4). However, cutoff values for these stages differ across studies. For significant fibrosis, CHI3L1 levels range between 60 and 90 ng/mL; for advanced fibrosis, levels range from 70 to 80 ng/mL; and for cirrhosis, levels range from 110 to 180 ng/mL. **(B)** Diagnostic models incorporating CHI3L1 have been developed for chronic hepatitis B (CHB) with significant fibrosis. In the context of non-alcoholic steatohepatitis (NASH) and associated fibrosis, several predictive models utilize CHI3L1. Notably, the NIS4® model includes YKL-40, miR-34a-5p, and alpha-macroglobulin, while the NIS2_+_™ model comprises YKL-40, miR-34a-5p, and sex as variables. For non-alcoholic fatty liver disease (NAFLD)/NASH with significant and advanced fibrosis, novel diagnostic models integrating CHI3L1 and other parameters are being actively explored. The figure was created with FigDraw (https://www.figdraw.com/#/).

In diagnosing NAFLD, CHI3L1 has been identified as a potential biomarker for NAFLD and diabetes-related liver fibrosis.^88^ However, many studies found that CHI3L1 alone may not be as effective as single biomarkers like CK-18^89^^,^^90^ or sSiglec-7^91^ in diagnosing NAFLD-related fibrosis. When CHI3L1 is combined with other parameters such as waist-to-height ratio, HA, procollagen–III–peptide, CK-18 neoepitope M65, and type IV collagen 7s, different diagnostic models can be established. These models are quite effective in diagnosing NAFLD with significant fibrosis (≥F2)^92^^,^^93^ and advanced fibrosis (≥F3).^94^ The AUROC of each model exceeds 0.80, depending on the formulas used. The diagnostic or predictive effects on hepatic fibrosis and at-risk NASH of CHI3L1 or CHI3L1-based models are presented in Figure 1.

### CHI3L1 serves as a biomarker for ALD-related fibrosis

CHI3L1 has been found to correlate with alcoholic cirrhosis.^95^ Later studies showed it can differentiate between advanced fibrosis (≥F3) and moderate fibrosis (≤F2) related to alcoholic liver disease (ALD).^96^ Moreover, Nøjgaard et al^97^ discovered that CHI3L1 and procollagen III N-terminal peptide (PIIINP) not only discriminate between different stages of ALD-related fibrosis but also reveal the prognosis of ALD by detecting plasma levels. The use of CHI3L1 or CHI3L1-based models as biomarkers for NAFLD or ALD-related fibrosis is summarized in Table 2.

**Table 2:** Diagnostic model based on CHI3L1 for NAFLD/NASH and related fibrosis.

Diagnostic model	Condition	Aim	Cutoff value	Efficacy	Comparison with other biomarkers	Ref.
NIS4® model	NASH at risk	Prediction	**Low cutoff value (not having at-risk NASH)**: Cutoff value = 0.36;**High cutoff value (having at-risk NASH):** Cutoff value = 0.63;	**Low cutoff value (not having at-risk NASH):** sensitivity = 0.815; Specificity = 0.630; NPV = 0.820; AUROC = 0.80 **High cutoff value (having at-risk NASH):** sensitivity = 0.507; Specificity = 0.871; PPV = 0.730	NA	86
NIS2_+_^TM^ model	NASH at risk	Prediction	**Low cutoff value (not having at-risk NASH):** Cutoff value = 0.46;**High cutoff value (having at-risk NASH):** Cutoff value = 0.68;	**Low cutoff value (not having at-risk NASH):** sensitivity = 0.850; = 0.610; NPV = 0.830; **High cutoff value (having at-risk NASH):** sensitivity = 0.620; specificity = 0.850; PPV = 0.770;	NIS2_+_^TM^ (AUROC = 0.813) > NIS4® (AUROC = 0.792) > AST (AUROC = 0.699) > FIB-4 (AUROC = 0.653)	87
Model consisting of CHI3L1, waist-to-height ratio, HA, procollagen–III–peptide, and CK18M65	NAFLD with significant fibrosis	Diagnosis	Cutoff value = 50;	sensitivity = 0.690; specificity = 0.818; NPV = 0.725; PPV = 0.791;	**NAFLD with significant fibrosis (≥F2):** The novel model (waist-to-height ratio, HA, procollagen–III–peptide, CHI3L1, and CK18M65) (AUROC = 0.829) > APRI (AUROC = 0.670) > FIB-4 (AUROC = 0.624) > NAFLD fibrosis score (NFS) (AUROC = 0.601) > BARD score (AUROC = 0.579)	92
Model consisting of CHI3LI, AST, type IV collagen, and LSM	NAFLD with significant fibrosis	Diagnosis	Cutoff value = -0.157	sensitivity = 0.696; Specificity = 0.917; NPV = 0.863; PPV = 0.800;	The diagnostic model (CHI3LI, AST, type IV collagen, and LSM) (AUROC = 0.864) > FIB-4 (AUROC = 0.726)	93
Model consisting of CHI3L1 and type IV collagen 7s	NAFLD with advanced fibrosis	Diagnosis	Cutoff value = 2.06;	Sensitivity: 0.850; Specificity: 0.786; NPV: 0.880; PPV: 0.739;	A new predictive model consisting of type IV collagen 7s and CHI3L1 (AUROC = 0.8763) > type IV collagen 7s (AUROC = 0.8458) > FIB-4 (AUROC = 0.7853) > CHI3L1 (AUROC = 0.7638) > HA (AUROC = 0.7527) > APRI (AUROC = 0.7429) > WFA ^+^ -M2BP (AUROC = 0.6953)	94

### CHI3L1 as a biomarker for HCC

AFP is commonly used as a biomarker to diagnose and predict the prognosis of HCC. CHI3L1 is another biomarker that may complement AFP, although some results question the utility of CHI3L1 in diagnosing HCC.^32^^,^^98^

Research has shown that serum levels of CHI3L1 are elevated in HCC patients, making it a valuable diagnostic biomarker for HCC. When combined with AFP, CHI3L1 improves diagnostic accuracy compared with using AFP alone (combination of CHI3L1 and AFP_AUROC_ = 0.8636 > AFP_AUROC_ = 0.8346).^99^ Moreover, CHI3L1 has been studied as a prognostic biomarker for HCC. According to the Kaplan–Meier survival curve, patients with positive CHI3L1 expression levels have a worse 1- and 3-year overall survival rate than those with negative expression (24.3 months versus 36.5 months, *p* < 0.001).^100^ Additionally, it is reported that the overall and recurrence-free survival of HCC patients who receive curative resection may be negatively correlated with serum CHI3L1 levels. High levels of CHI3L1 within six months after curative resection may indicate poor prognosis, with a hazard ratio of 3.003 (*p* = 0.009).^101^ Similarly, CHI3L1 can also predict the prognosis of HCC patients who received transhepatic arterial chemotherapy and embolization. Elevated CHI3L1 levels indicate poor prognosis, with a mortality prediction efficacy indicated by AUROC of 0.629 (*p* = 0.005), superior to AFP (AUROC = 0.569).^102^

Besides diagnosis and prognosis prediction, CHI3L1 could identify HCC patients at risk and predict disease development. In Egyptian patients who have sustained virological response, the single-nucleotide polymorphisms of CHI3L1 (rs880633 and rs597533) have indicated susceptibility to HCC.^103^

Taken together, CHI3L1 has the potential to indicate risk for NASH and HCC. Additionally, CHI3L1 could serve as a diagnostic biomarker for hepatitis-related fibrosis, ALD-related fibrosis, and HCC. Combining CHI3L1 with other parameters may improve the diagnostic performance for hepatitis- and NASH-related fibrosis.

### CHI3L1 as a biomarker for other liver diseases

CHI3L1 levels have been found to increase in pregnant patients with intrahepatic cholestasis.^104^ Additionally, CHI3L1 levels increase in portal hypertension and are associated with hepatic function.^105^^,^^106^ These findings may inspire the applications of CHI3L1 in diagnosing other liver diseases.

### Regulation of liver disease pathophysiology by CHI3L1: Mechanisms and clinical implications

#### CHI3L1 in HCC: mechanisms of tumorigenesis and metastasis

HCC is the third most common cancer-related cause of death globally.^107^ Identifying key targets and pathways in HCC is crucial for drug development.

CHI3L1 has been recognized as a poor prognostic biomarker for HCC and is involved in promoting HCC metastasis.^108^ Mechanistically, CHI3L1 promotes HCC metastasis by activating transforming growth factor β (TGF-β), which phosphorylates the SMAD family (SMAD2 and SMAD3) and promotes HCC proliferation, invasion, and metastasis.^109^ However, the exact pathway by which CHI3L1 induces HCC has not yet been confirmed. Sarcopenia, commonly found in the elderly, is a risk factor for HCC. Given the aging population, sarcopenia-related HCC requires attention. Research has shown that CHI3L1 secreted by muscle cells can alleviate sarcopenia by up-regulating the tumor necrosis factor (TNF)-α/TNF-R1 signaling pathway. However, CHI3L1 can also induce the accumulation of lipid peroxides, promoting HCC progression.^110^

Studies have revealed that CHI3L1 could contribute to other cancer types. For instance, CHI3L1 derived from M2 macrophages could interact with interleukin-13 receptor α2 chain (IL-13 Rα2), activating mitogen-activated protein kinase (MAPK) signaling pathway and promoting the metastasis of breast cancer.^111^ Moreover, it could activate MAF/cytotoxic T lymphocyte-associated antigen 4 (CTLA4) signaling pathway, which has contributed to the immune escape by attenuating CD8^+^ T cell cytotoxicity.^112^ In colon cancer, CHI3L1 could decrease the expression of p53 and increase the expression of endothelial growth factor receptor (EGFR), which resulted in the proliferation of cancer cells. Immunotherapy is a conventional treatment for solid tumors, and CHI3L1 has played a role in the T cell-based cancer immunotherapies. It could promote melanoma progression by activating programmed cell death 1 (PD-1)/programmed death ligand 1 (PD-L1) axis.^113^ A study has found that CHI3L1 could activate various signaling pathways, including protein kinase B (AKT), β-catenin, and nuclear factor kappa B (NF-κB), and increase the number of regulatory T cells, stimulating the tumor microenvironment.^25^ In 2023, Tarek et al^11^ have discovered that CHI3L1 could hinder T cell infiltration through recruiting neutrophils and forming neutrophil extracellular traps. Given the potential role of CHI3L1 in T cell-based immunotherapies in various tumor types,^114^, ^115^, ^116^ it is speculated that targeting CHI3L1 may promote anti-tumor immunity in HCC.

There is a scarcity of research on the mechanisms by which CHI3L1 contributes to HCC, and more efforts are needed to elucidate this. Developing an antibody that targets CHI3L1 may be a strategy for treating HCC.

### CHI3L1 and hepatic fibrosis: role in fibrogenesis and clinical implications

Hepatic fibrosis can lead to cirrhosis and HCC, making it crucial to find effective treatments. Unfortunately, there are currently no targeted drugs for hepatic fibrosis available on the market. Treating hepatic fibrosis involves identifying specific causes such as viruses, ethanol, or parasites, addressing the underlying issues, and stopping the activation of hepatic stellate cells. Pinpointing crucial targets in hepatic fibrosis will be instrumental in finding a solution.

Recent studies suggest that CHI3L1 plays an essential role in hepatic fibrosis. Cheng et al^117^ found that HCV infection caused an increase in oxidative stress *in vitro*, which activated the MAPK and TNF-α pathways. This, in turn, triggered the NF-κB signaling pathway, leading to the production of CHI3L1 in hepatocytes. CHI3L1 stimulates hepatic parenchymal cells and hepatic stellate cells, resulting in the production of fibrosis markers such as TGF-β1 and vascular endothelial growth factor A (VEGFA).

Besides hepatic stellate cells, hepatic macrophages are also involved in hepatic fibrosis, and they may activate the Akt signaling pathway and inhibit the apoptosis of macrophages in the liver through the Fas signaling pathway, both of which result in hepatic fibrosis.^118^

### CHI3L1 in liver injury: involvement in acute and chronic liver damage

Acute liver failure is a life-threatening condition caused by various factors, including ischemia/reperfusion, coagulation, endotoxins, overactive immune responses, as well as extrinsic factors such as drug abuse and alcohol addiction.

Acetaminophen overdose is a significant cause of acute liver failure in certain developed countries and regions.^119^^,^^120^ While N-acetylcysteine can treat acetaminophen-induced liver injury, its effectiveness is limited to early administration. Unfortunately, around one-third of patients do not respond to N-acetylcysteine treatment.^121^ As a result, there is a critical need to investigate alternative, more effective drugs or strategies for managing acetaminophen-induced liver injury.

A recent study revealed the crucial role of CHI3L1 in promoting intrahepatic vascular coagulation in a concanavalin A-induced liver injury model.^122^ Tissue factor (TF) has been recognized as a key target for intrahepatic vascular coagulation. Lun et al^123^ found that CHI3L1 could activate TF and its target, protease-activated receptor 1 (PAR1), facilitating chemokine expression, inflammatory cell accumulation, and liver injury. Concanavalin A causes hepatic immune injury, a common mechanism of liver damage.^124^ Researchers found that CHI3L1, derived from mesenchymal stem cells (MSC), could inhibit T cell activation and proliferation, and alleviate concanavalin A-induced liver injury by activating the peroxisome proliferator-activated receptor-delta (PPARδ)/signal transduction and activator of transcription 1/3 (STAT1/3) signaling pathway.^125^ Additionally, it was discovered that CHI3L1 interacts with CD44, its receptor on liver macrophages, prompting the interaction between podoplanin/C-type lectin-like receptor 2 (Clec-2) and inducing platelet recruitment. This leads to inflammation, intrahepatic coagulation, and hepatocyte damage. Therefore, this study provides insights into the mechanism of acetaminophen-induced liver injury and identifies CHI3L1 as a potential target for treating this liver injury.^126^ Ethanol addiction is a widespread concern that can lead to the development of ALD. For treating ALD, CHI3L1 has proven to be a promising candidate. In mouse models, CHI3L1 effectively inhibited hepatic lipogenesis and inflammation caused by an ethanol-containing diet, making it a promising target for ALD therapy.^127^

During liver transplantation, ischemia/reperfusion is a common process that can cause liver injury and result in poor survival rates. Ischemia/reperfusion-induced injury can be mitigated by CHI3L1 secreted by M2 macrophages during adaptation, restoring hepatic metabolism and mitochondrial function.^128^ Additionally, studies have linked the activation of MMP and CHI3L1 after ischemia/reperfusion to profibrotic and proinflammatory outcomes of liver injury. Fortunately, bortezomib can inhibit their expression and mitigate these outcomes.^129^

Liver injury can also occur due to brain death, which increases oxidative stress and induces hepatocyte apoptosis.^130^ Recent research indicates that CHI3L1 plays a crucial role in this process by inducing M1 polarization of macrophages and exacerbating hepatic inflammation. Additionally, it activates the proteinase-activated receptor 2 (PAR2)/c-Jun N-terminal kinase (JNK)/caspase-3 signaling pathway, resulting in hepatocyte apoptosis.^131^ Similarly, lipopolysaccharide, a common endotoxin, can induce M2 polarization of macrophages, which is mitigated by the knockout of CHI3L1. This implies that CHI3L1 may facilitate the development of acute liver injury through immune dysfunction.^132^ In a NOD-like receptor protein 3 (NLRP3)-induced inflammatory model, the deficiency of breast regression protein 39 (BRP39) contributed to increased circulatory neutrophils and reduced immune cells in the liver, alleviating hepatic inflammation.^133^ It has been shown that CHI3L1 expression parallels cytokine-induced liver inflammation or fibrosis, indicating that CHI3L1 may serve as an intervention target for acute or chronic liver injury and related inflammation and fibrosis.^134^

However, another study led by Li et al^135^ found that CHI3L1 may have a protective effect against overactive inflammation in acute graft-versus-host disease by relieving the inhibition of regulatory T cells.

### CHI3L1 in NAFLD: mechanisms and potential therapeutic target

CHI3L1 positively impacts lipogenesis and exhibits anti-NAFLD effects. The knockout of CHI3L1 significantly reduces lipid accumulation and improves insulin resistance in NAFLD through the pAKT/pGSK-3β (pERK) signaling pathway.^136^ In a choline-deficient, l-amino acid-defined, high-fat diet (CDAA-HFAT)-induced NASH model, CHI3L1 levels increased in macrophages. CHI3L1 could combine with IL-13 receptor α2 (IL13Rα2) to activate hepatic stellate cells, producing collagen and inducing hepatic fibrosis. This finding suggests that CHI3L1 may serve as a potential target for NASH-related fibrosis.^137^

**Figure 2:**
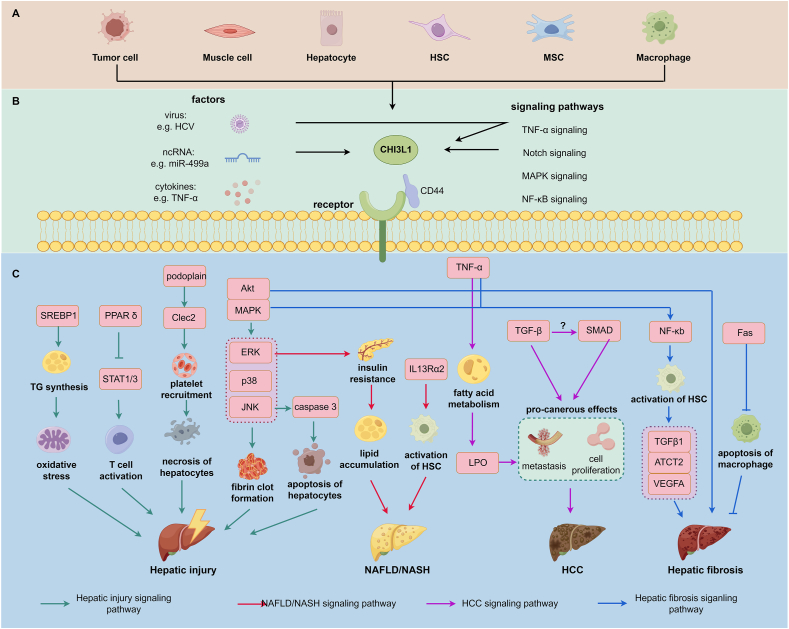
The regulatory role of CHI3L1 on various liver diseases. **(A)** Cellular sources of CHI3L1. CHI3L1 is secreted by a variety of cells, including tumor cells, muscle cells, hepatocytes, hepatic stellate cells (HSCs), mesenchymal stem cells (MSCs), and macrophages. **(B)** Regulation of CHI3L1 expression. The production of CHI3L1 is influenced by multiple factors such as viral infections, non-coding RNAs (ncRNAs) like miR-499a, and cytokines such as TNF-α and IFN-γ. Additionally, CHI3L1 expression is modulated by several signaling pathways, including the TNF-α, Notch, MAPK, and NF-κB pathways. There is also significant crosstalk between these factors and signaling pathways, which synergistically regulate CHI3L1 expression. **(C)** Mechanisms of CHI3L1 in liver diseases. CHI3L1 can bind to CD44 or other receptors to enter cells, subsequently regulating hepatic injury through various pathophysiological processes. These processes include the elevation of oxidative stress, T cell activation, fibrin clot formation, apoptosis, and necrosis, mediated by pathways such as PPARδ, Akt/MAPK, and caspase signaling. In the context of non-alcoholic fatty liver disease (NAFLD)/non-alcoholic steatohepatitis (NASH), CHI3L1 modulates lipogenesis and fibrosis through the ERK and IR13Rα2-dependent pathways, respectively. Moreover, CHI3L1 may contribute to the development of hepatocellular carcinoma (HCC) via the TGF-β and SMAD signaling pathways. During hepatic fibrosis, CHI3L1 is regulated by NF-κB, Akt, and Fas signaling pathways, which stimulate the activation of HSCs and macrophages, thereby inducing liver fibrosis. The figure was created with FigDraw (https://www.figdraw.com/#/).

The regulatory roles of CHI3L1 in various liver diseases are summarized in Table 3. The overall signaling pathways that CHI3L1 has mediated in hepatic injury, NAFLD (NASH), HCC, and hepatic fibrosis are shown in Figure 2.

**Table 3:** CHI3L1 in the regulation of various liver diseases.

Disease	Source of CHI3L1	Detection method of CHI3L1	Conclusion	Ref.
HCC	Human liver cancer cell lines (HepG2 and Bel7404)	RT-PCR, WB, IF	CHI3L1 promotes HCC by regulating cell proliferation, invasion and metastasis mediated by TGF-β signaling pathway and SMAD signaling pathway.	109
Sarcopenia related HCC	Muscle cells	ELISA, WB	CHI3L1 secreted by muscle tissues alleviates sarcopenia through TNF-α/TNF-R1 signaling, but it may increase LPO to accumulate to promote HCC progression.	110
CHC with fibrosis	Hepatic parenchymal cells	RT-PCR, western blotting (WB)	HCV interacts with CHI3L1 to induce the generation of profibrogenic cytokines, resulting in HCV related fibrosis.	117
Varius etiologies induced liver fibrosis	Hepatocytes, hepatic stellate cells (HSC)	RT-PCR, ELISA, IF	CHI3L1 stimulates HSCs to proliferate and activate to generate fibrosis related biomarkers.	34
Intrahepatic activation of coagulation (IAOC) related liver injury	Murine liver	RT-PCR, ELISA	CHI3L1 induces IAOC through tissue factor (TF) mediated fibrin clot formation dependent by MAPK signaling pathway.	122
Immune-mediated liver injury	Human umbilical cord MSCs (hUC-MSCs)	NA	CHI3L1 secreted by hUC-MSCs inhibits immune mediated liver injury by upregulating PPARδ, which suppresses STAT1/3 modulated T cell activation and proinflammatory cytokines release.	125
Acetaminophen-induced liver injury	Murine and human liver, serum	ELISA, RT-PCR, IHC and IF	CHI3L1 binds with CD44 on macrophages to induce podoplanin and Clec2 expression, which mediated platelet recruitment and liver damage.	126
Ethanol-induced liver injury	External transfection	NA	Inhibition of CHI3L1 suppresses ethanol-induced liver injury through inhibiting SRBP1 modulated TG synthesis, which is associated with inflammation of liver.	127
Liver ischemia/reperfusion injury	Rat liver	WB	Transplantation induced I/R has increased the expression of CHI3L1, which may promote the generation of pro-fibrotic and pro-inflammatory cytokine release and cause liver injury.	129
Brain death-induced liver injury	Rat liver, liver macrophages (THP-1) and hepatocytes (LO-2 cell)	RT-PCR, WB, IHC and IF	CHI3L1 leads to the apoptosis of hepatocytes by PAR2/JNK/caspase 3 signaling pathway.	131
NAFLD	Murine and human liver tissues	RT-PCR, ELISA, IHC	CHI3L1 contributes to insulin resistance and lipid accumulation in liver by Akt signaling pathway.	136

## Conclusion and future perspective

CHI3L1's potential as a diagnostic and prognostic biomarker for liver diseases is increasingly recognized. As a biomarker, CHI3L1 demonstrates high sensitivity and specificity in detecting liver fibrosis, NAFLD, ALD, and HCC. Its levels are closely associated with disease severity, making it an attractive tool for early detection and staging of liver diseases. In particular, CHI3L1 could serve as a non-invasive alternative to liver biopsy, aiding in the monitoring of disease progression and response to therapeutic interventions. The combination of CHI3L1 with other biomarkers holds promise for improving diagnostic accuracy, creating more reliable models for predicting disease outcomes, and guiding treatment decisions.

**Figure 3:**
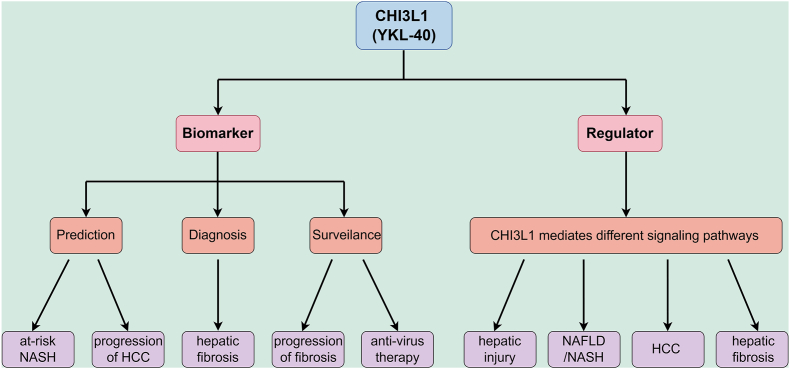
CHI3L1's dual roles as a biomarker and regulator for various liver diseases.

In addition, we have extensively discussed the multifaceted role of CHI3L1 in liver diseases, focusing on its involvement in liver fibrosis, NAFLD, ALD, hepatitis, and HCC. We have highlighted its significant contribution to liver disease progression through mechanisms such as inflammation, fibrosis, and tumorigenesis. As a glycoprotein secreted by activated hepatic stellate cells, macrophages, and other liver cells, CHI3L1 plays a critical role in the pathological processes of these diseases. Moreover, its involvement in immune regulation, tissue remodeling, and extracellular matrix deposition positions it as a crucial player in liver disease development. Given its central role in promoting liver inflammation, fibrosis, and carcinogenesis, targeting CHI3L1 through inhibition or neutralization could offer new treatment avenues, particularly for patients with chronic liver diseases and liver cancer. By blocking CHI3L1's pro-fibrotic and inflammatory effects, it may be possible to halt or reverse the progression of fibrosis and prevent the development of HCC. This therapeutic approach would require further exploration in clinical trials to determine its efficacy and safety in human populations. The role of CHI3L1 as the biomarker and regulator is depicted in Figure 3.

However, the clinical translation of CHI3L1 as a biomarker and therapeutic target faces several challenges. Standardized assays for measuring CHI3L1 levels must be developed to ensure reproducibility and reliability across different clinical settings. Large-scale clinical studies are essential to validate CHI3L1 as a biomarker for early diagnosis and prognosis in liver diseases, especially in diverse patient populations.

Further research is needed to fully understand the molecular mechanisms underlying CHI3L1's role in liver disease and its interactions within the complex liver microenvironment. CHI3L1's interactions with other liver-specific signaling pathways and its role in liver regeneration will provide insights into its broader implications in liver health. Future therapeutic strategies targeting CHI3L1 may include small-molecule inhibitors, monoclonal antibodies, or gene therapy approaches. These therapeutic interventions could complement existing treatments for liver diseases and offer new hope for patients with limited options.

In conclusion, CHI3L1 represents a promising biomarker and therapeutic target for liver diseases. Continued research and clinical trials will be crucial to unlocking its full potential in improving both diagnostic capabilities and treatment outcomes, ultimately paving the way for more effective management of liver diseases in clinical practice.

## CRediT authorship contribution statement

**Chao Tian:** Writing – review & editing, Writing – original draft, Visualization, Software, Investigation, Conceptualization. **Shizhou Deng:** Writing – review & editing, Validation, Supervision, Funding acquisition. **Ming Yang:** Writing – review & editing, Validation, Supervision. **Baochen Bai:** Writing – review & editing, Visualization. **Lai Wei:** Writing – review & editing, Validation, Supervision, Project administration, Funding acquisition.

## Funding

This work was supported by the 10.13039/501100012166National Key R&D Program of China (No. 2022YFA1303804).

## Conflict of interests

The authors declared no conflict of interests.
